# Local recording of biological magnetic fields using Giant Magneto Resistance-based micro-probes

**DOI:** 10.1038/srep39330

**Published:** 2016-12-19

**Authors:** Francesca Barbieri, Vincent Trauchessec, Laure Caruso, Josué Trejo-Rosillo, Bartosz Telenczuk, Elodie Paul, Thierry Bal, Alain Destexhe, Claude Fermon, Myriam Pannetier-Lecoeur, Gilles Ouanounou

**Affiliations:** 1 Unité de Neuroscience, Information et Complexité (UNIC), FRE CNRS 3693, Gif-sur-Yvette, France; 2 Center for Neurophysics, Physiology and Pathologies, UMR CNRS 8119, Université Paris Descartes, Paris, France; 3 SPEC, CEA, CNRS, Université Paris-Saclay, CEA, Saclay 91191 Gif-sur-Yvette Cedex, France; 4 European Institute for Theoretical Neuroscience (EITN), Paris, France

## Abstract

The electrical activity of brain, heart and skeletal muscles generates magnetic fields but these are recordable only macroscopically, such as in magnetoencephalography, which is used to map neuronal activity at the brain scale. At the local scale, magnetic fields recordings are still pending because of the lack of tools that can come in contact with living tissues. Here we present bio-compatible sensors based on Giant Magneto-Resistance (GMR) spin electronics. We show on a mouse muscle *in vitro*, using electrophysiology and computational modeling, that this technology permits simultaneous local recordings of the magnetic fields from action potentials. The sensitivity of this type of sensor is almost size independent, allowing the miniaturization and shaping required for *in vivo/vitro* magnetophysiology. GMR-based technology can constitute the magnetic counterpart of microelectrodes in electrophysiology, and might represent a new fundamental tool to investigate the local sources of neuronal magnetic activity.

Electrophysiology offers a wide range of techniques to record electrical activity spanning from the microscale (single ion channel and intracellular recordings), to the mesoscale (Local Field Potential), up to the brain macroscale (Electroencephalography). Thanks to the experimental access to the local measurements, the nature of the sources underlying the extracellular electrical signal at all scales is identified and its underlying biophysics is well understood^1^. Any transmembrane current, irrespective of its origin (fast action potential, synaptic currents, slow fluctuations in glia) participates to the genesis of extracellular potentials.

Contrary to electric fields, magnetic fields (MFs) have not been measured at many scales. Indeed, the Superconducting QUantum Interference Device (SQUID) magnetometers used for magnetoencephalography (MEG)^2^, even if sensing MFs of the order of fT at centimeters from the source, cannot be miniaturized and work at helium or liquid nitrogen temperature, which precludes any possible contact with living tissues. Therefore, due to the lack of tools for local recordings, the interpretation of the MEG signal is essentially based on untested hypotheses concerning the nature of the local sources^3^. Synaptic activity in dendrites generates dipoles driving axial currents, considered as the main local sources of the MF^4^. It then appears evident that the direct experimental access to the local sources of the signal would constitute an essential tool to investigate and interpret MEG signals.

Only recently, some attempts have been made to build new technologies able to detect biological MFs at the local scale. Barry *et al*.^5^, used the nitrogen-vacancy (NV) quantum defects in diamond to measure the MF produced by action potentials (APs) in the squid and worm giant axons. We explore here an alternative technique, based on the giant magneto-resistance (GMR) effect^6^. GMR-based sensors have been commonly used in the industry and work at room temperature. They are made of thin-film structures of alternating ferromagnetic and non-magnetic layers, whose electrical resistance depends on the applied MF. We took advantage of this technology to build magnetic probes adapted to biological preparations. GMR-based sensors are highly malleable in shape, can be miniaturized at the micro-scale without sensitivity loss^7^, and, working at room temperature, can be placed in contact or potentially penetrate the living tissue, permitting highly local recordings both *in vitro* and *in vivo*. Simply electrically supplied with two wires, its handling is as simple as for an electrode.

In order to validate the use of the GMR-based technology in biology, we focused on a simple biological structure where the expected magnetic pattern was easily predictable. We chose the mouse skeletal soleus muscle for the simplicity of its organization and for the synchronicity of the electrical activity among the muscle fibers. Indeed, skeletal muscles are composed by set of parallel excitable cables, innervated at their center by a single excitatory cholinergic synapse. The synaptic transmission is optimal in the sense that each single nerve stimulation induces a post-synaptic AP^8^,^9^. Furthermore, synapses are well aligned at the center of the muscle, resulting in a strong synchronicity among the fibers APs. Robustness of the synaptic transmission allows averaging over trials, and parallel organization and synchronicity of the APs maximize MF summation across fibers. The fact that face skeletal muscles activity is perceived in MEG^4^,^10^,^11^ and can be of several order of magnitude higher than the putative brain signal in the same frequency range further supports the idea that muscles individually produce large MFs, thus constituting a convenient model for a first test of the GMR technology in biology.

## Theoretical framework

**Figure 1:**
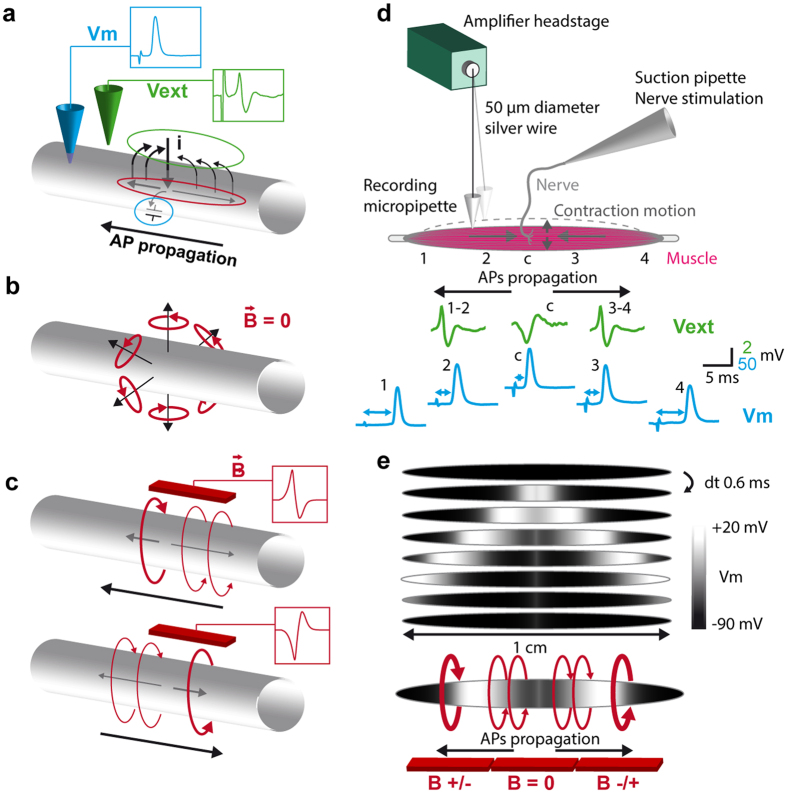
Electrophysiology. (**a**) Transmembrane and axial currents in an active zone of an excitable cable. Charge of the membrane capacitor is the quantity measured with an intracellular pipette (blue circle). An extracellular electrode measures the effect of the transmembrane currents on the external potential (green circle). Axial currents (red circle) are not directly measured with classical electrophysiology. (**b**) Theoretical magnetic fields associated to transmembrane currents regularly distributed along the circumference of a cable. Seen from a certain distance, contributions of the transmembrane currents to the net magnetic field cancel each other. (**c**) Theoretical magnetic field associated to axial currents flowing in front and back of an action-potential in an excitable cable. Sign of the magnetic field depends on the current direction. (**d**) Scheme depicts the floating electrode technique used for intra and extracellular recordings in the contracting muscle. The extreme tip of a glass micropipette is used as electrode, and hangs at the extremity of a free moving silver wire connected by its opposite end to the amplifier headstage. The absence of pipette holder allows the free moving of the system and the intracellular recordings during contraction. Green traces represent the extracellular potential measured in the synaptic region, and on both sides of the muscle, when the nerve is electrically stimulated. Blue traces represent the intracellular recording of the muscle AP, in the synaptic region and in different positions on both sides of the central region. (**e**) Gray scale representation of the simulated membrane potential at different times showing the internal gradients at the front and the back of the two active zones. Bottom scheme represents the expected magnetic fields associated to the muscle AP.

In excitable cables such as axons or muscle fibers, Na^+^ enter the cable through the voltage-gated Na^+^ channels. Part of these electrical charges raises the membrane potential while another part leaks through passive and active K^+^ and Cl^−^ conductances, and establishes a loop with the source current in the extracellular space (Fig. 1a). The level of capacitance charge, i.e. the membrane potential, is the physical quantity directly measured with an intracellular microelectrode (Fig. 1a, delineated with a blue ellipse). Leakage of the electrical charges along the cable is responsible of the local aspect of the membrane depolarization. In the extracellular space, the local effects of inward and outward transmembrane currents on the external potential (Fig. 1a, green ellipse) can be measured with an extracellular microelectrode. Interestingly, the intra-cytoplasmic flows of ions along the internal potential gradient constitute axial currents at the front and the back of the active zone (Fig. 1a, red ellipse) and are not directly measured with classical electrophysiological techniques.

According to the theory of electromagnetism, any current generates a MF. However, transmembrane currents are not considered to significantly contribute to MEG signal^4^,^12^, since the homogenous distribution of ionic channels along the circumference of neurites, axons and cell body should result in the cancelation of the contributions of transmembrane currents to the net MF (Fig. 1b) when measured from large distance compared to cable diameter. Therefore, only axial currents in cables are expected to participate to the net MF, whose sign will depend on the axial current direction (Fig. 1c). The return currents flowing in the extracellular space are also expected to generate MFs, but in opposite direction, partially screening the intracellular source.

## Results

### Skeletal muscle electrophysiology

Electrical activity of the neuromuscular system has been studied for decades and is well documented. However, because the MF depends on the AP shape and propagation speed, we performed an electrophysiological characterization of the mouse soleus nerve-muscle preparation in our specific experimental conditions. Conventional intracellular recordings are not possible in mouse skeletal muscle due to macroscopic motion during contraction. In order to maintain the intracellular recording in the moving tissue, we used a “floating electrode” technique inspired from refs 13 and 14 (Fig. 1d, see Methods).

When the nerve is introduced into a suction pipette, an AP can be triggered in all the axons of the nerve with a voltage drop applied in the suction pipette. The subsequent synaptic release of the acetylcholine neurotransmitter and the nicotinic receptors activation is able to trigger the muscle AP in all the muscle fibers synchronously. We proceeded to the intracellular recording of the membrane potential at different locations, spaced by 3 mm and covering the muscle 1 cm length, as well as at the synaptic central region. The shortest delay (≈2 ms) was found in the central region, and was mainly due to the synaptic delay. At 1.5 mm and 4.5 mm from the synaptic region, delays of ≈2.7 and 5 ms respectively were found on both sides of the synaptic zone. These recordings show that the muscle AP rises in the central region of the muscle, and splits in two APs that propagate in opposite directions towards the extremities with a propagation speed of 2–3 m.s^−1^. On the two sides of the central synaptic region, the extracellular recordings exhibit the expected tri-phasic pattern corresponding to the effects on the external potential of the positive transmembrane currents on the front and the back of the active zone and of the negative transmembrane currents at the active zone. In the central region, the extracellular potential shows only two phases, corresponding to the effects of the negative transmembrane currents followed by the effects of the positive currents on the back of the two active zones. Figure 1e, based on the simulations described in the last result section, illustrates with a gray scale the propagation in opposite directions of the two muscle APs.

Three regions of interest thus emerged from the electrophysiological characterization of our experimental model: a synaptic central region where the AP rises, and the two sides of the muscle where the APs propagate in opposite direction and where MFs are expected with opposite sign (Fig. 1e, bottom scheme).

### GMR-based sensors

**Figure 2:**
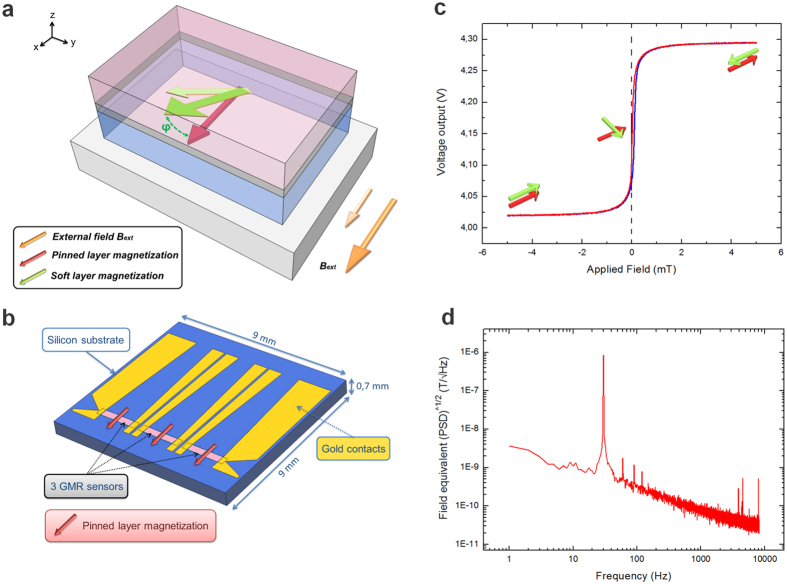
GMR sensors. (**a**) Schematic representation of the trilayer structure constituting the spin valve deposited on a silicon substrate (light gray). The pinned layer (light blue) has a fixed magnetization along a specific direction (red arrow). A thin copper layer (dark gray) decouples the pinned layer from the free layer (light pink). An external field applied in the plane of the stack rotates the free layer magnetization, which forms an angle ϕ with the pinned layer, according to the field strength (illustrated with light and dark green arrows according to the external field strength in light and dark orange). The resistance of the stack varies as function of the angle between the free and the pinned layer. (**b**) GMR sensor configuration. The sensor comprises three segments (light pink) with pinned layer magnetization perpendicular to the segment length (red arrows). Each segment is contacted through two lines of gold/titanium (yellow). (**c**) Voltage variation of a single segment as function of an in-plane field applied along the pinned layer magnetization. Red (blue) curve is obtained when the field is swept from large negative (positive) values to large positive (negative) values. (**d**) Field equivalent noise (square root of the voltage power spectrum density (PSD) divided by the sensitivity), of the segments in a magnetically shielded room. The calibration from V/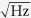 to T/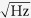 is made by using a calibrated signal at 30 Hz.

First discovered in 1988^6^, the Giant Magneto-Resistance (GMR) effect is based on the difference of conductivity for electrons in ferromagnetic materials, depending on the relative orientation between their spins and the magnetization of the layer. We used a sensor based on the GMR effect in a trilayer structure, called a spin valve^15^. A spin valve sensor is made of a magnetic layer exhibiting a strong coercivity (*hard layer*) separated from a magnetic layer with a very low coercivity (*soft layer*) by a thin metallic spacer (Fig. 2a). The magnetization of the soft magnetic layer can easily rotate along an in-plane applied field, whereas the hard layer keeps its initial orientation as long as the applied field is lower than its coercivity. The resistance of the whole stack varies with the angle between the magnetization axes of the two layers. GMR sensors exhibit a sensitivity (expressed as a variation of the output voltage for a given magnetic induction in T and normalized at 1 V) of typically 50 V/T in the field operating range (see Methods). Unlike flux sensors used in bio-magnetism (SQUID for example), GMR sensors can be miniaturized and are able to operate at room temperature.

Considering the size of the soleus muscle (1 cm-long), we adapted the shape of our sensors by designing three aligned spin valves of 1, 7 mm * 400 μm (Fig. 2b), oriented in such a way that they would be sensitive to a MF generated by an axial current flowing in the length of the muscle fibers. These sensors face the three different zones defined by their specific electrophysiological activities. Each sensor response corresponds to the MF averaged on the sensor length. The GMR stack, deposited on a 700 μm thick silicon substrate, was patterned by laser lithography, and then etched by an ion beam. The gold contacts were deposited by evaporation at the edges of the three segments. Each of the sensors exhibits a resistance of 80 Ω and was fed by a direct current of 20 mA. The change in resistance induced by a magnetic field of 1 nT will lead to an output voltage variation of about 80 nV across the sensor. The sensors were passivated first by a bilayer of Al_2_O_3_/Si_3_N_4_ deposited by sputtering and second a thin layer of polymer resin. That double passivation avoids electrical contamination from the extracellular potential variations and ensures insulation of the sensors submerged in the physiological saline solution.

The sensor output voltage as a function of an in-plane applied field B exhibits a linear variation around zero field whose slope defines the sensitivity (Fig. 2c). The MF detection capability of the sensor over frequency can be estimated by recording the power spectrum density in a magnetically shielded room while applying a calibration signal of known amplitude and frequency (Fig. 2d). A GMR sensor being a small volume conductor, it exhibits 1/f noise at low frequency^16^,^17^ and reaches the thermal noise bottom limit for f > 5 kHz. In the frequency range of physiological MFs, this 1/f noise gives the field detection limit of the sensor. From these noise measurements, one may estimate the integrated magnetic noise over the working bandwidth (1.2 kHz) to be of 3.5 nT *rms*. A signal-to noise ratio (SNR) of 2 would therefore require 50 averaging to detect a signal of 1nT peak-to-peak (see Methods).

### Magnetic recordings

**Figure 3:**
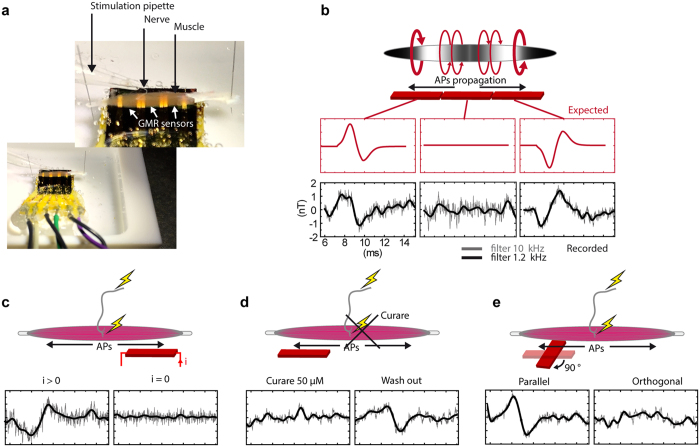
Magnetic recordings. (**a**) Photography of the recording chamber, with the muscle held by its tendons on the top of the magnetic probe, and with the nerf sucked into a stimulating glass pipette. (**b**) Expected and recorded magnetic signal on the three GMR-sensors after electrical stimulation of the nerve. Traces are averages over 500 trials, filtered with a low pass filter at 10 (gray traces) or 1.2 kHz (black traces). (**c**) In absence of feeding current in the GMR sensor, the absence of electrical signal shows the quality of the electrical insulation and demonstrates that the recorded signal is purely magnetic. (**d**) MF following nerve stimulation in control conditions, in presence of the reversible synaptic receptors blocker curare, and after wash out. (**e**) Juxtaposition of the MFs recorded with the muscle held successively orthogonal and parallel with respect to the probe sensitivity orientation.

The soleus mouse nerve-muscle preparation was placed on the probe and hold with minutien pins through the tendons (Fig. 3a). The three aligned segments constituting the probe, each 1.7 mm long, cover the total length of the muscle and allow simultaneous recordings on each third of the muscle that correspond to the three regions of interest mentioned in section “Skeletal muscle electrophysiology”. As shown in Fig. 3b, modeling predicted a bi-phasic pattern for the MF with opposite sign at the two external segments, and flat at the central segment. Figure 3b shows the MFs recorded simultaneously by the three segments of the probe. As expected, the MF detected by the segment 1 and 3 of the probe displays a biphasic shape which matches the predictions, with a total duration of 4.8 ms and a temporal delay between the two peaks of 2 ms. Magnetic detection being vectorial, the signs of the signals were in agreement with the axial currents directions. The opposite polarity of these two signals thus gives a first confirmation that it is indeed the axial currents direction that determines the sign of the MF. At the central region (segment 2), the MFs generated by the two APs propagating in opposite direction are simultaneously perceived by the same probe segment, and, because of the spatial averaging performed by the probe, this results in the cancellation of the different opposite contributions.

The peak-to-peak amplitude of the signal, averaged over 500 events in Fig. 2b, was 2.7 nT. This value of amplitude was consistent among the nerve-muscle preparations (mean ± SD = 2.8 ± 0.7 nT, n = 6 muscles from different mice). As it will be shown in the section ‘Modeling’, the amplitude of the signal depends not only on the amplitude of the axial currents and the number of muscle fibers, but also on the screening effects of the return currents in the extracellular space. In Fig. 3b, the signal was filtered at the acquisition with a 10 kHz cutting frequency (gray traces), and the signal to noise ratio (SNR) of the averaged signal (n = 500 events) was 7.5 on the segment 1 and 10.15 on the segment 3. With an additional off-line filtering, with a cutting frequency of 1.2 kHz (black traces in Fig. 3b), the SNR raised to 9.56 and 15.3 for the segments 1 and 3 respectively. The SNR evolving as a function of 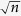, 10 events (SNR > 2) was the threshold value for magnetic AP detection.

The transmembrane currents being able to raise the potential of the extracellular medium by several mV, and the MF being measured through the polarization of the GMR sensor, the electrical insulation of the GMR sensor and of the gold connectors is critical to ensure a strictly magnetic measurement. The quality of the insulation, and the absence of electrical contamination of the signal, could be controlled with nerve stimulations (n = 500) in the absence of feeding current in the GMR sensor (Fig. 3c). Furthermore, in order to control that the recorded magnetic signal was due to the muscle activity only, we used the nicotinic receptors antagonist curare to prevent the muscle APs while preserving nerve activity (Fig. 3d). Wash out of the curare antagonist restored the muscle activity and the characteristic magnetic signature (Fig. 3d). Finally, we controlled that the magnetic signature associated with the muscle AP was not visible when the muscle was placed orthogonally to the GMR sensor (Fig. 3e). The absence of detectable signal further confirms the assumption that the transmembrane currents have a negligible contribution to the MF, which is mainly due to the axial currents flowing in the direction of the fibers.

### Modeling

**Figure 4:**
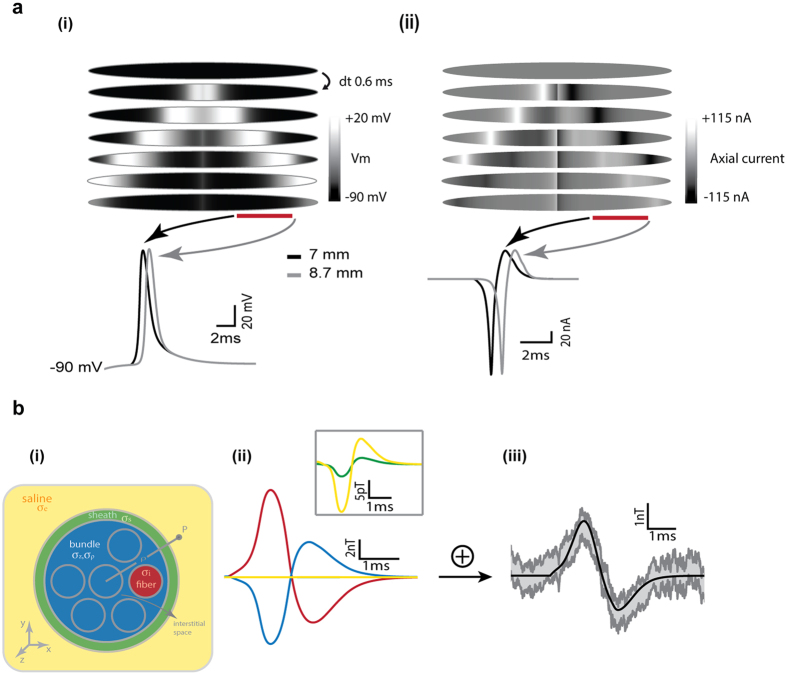
Modeling versus experiments. (**a**) (i) Evolution of the membrane potential along the muscle fiber for successive times. The time evolution of the membrane potential during the AP in two different sites of the fiber corresponding to the two ends of the probe is depicted on the bottom. The time lapse between the two AP peak is 0.7 ms, corresponding to an AP speed of 2.4 m/s. (ii) Same as in (i) but for the axial currents generated by the membrane potential gradients along the fiber. (**b**) (i) Scheme of the muscle section with the different sub-regions depicted in different colors. (ii) The components of the calculated magnetic field due to the currents flowing in the different regions depicted in (i): the fibers (red), the bundle (blue) and the sheath and bath saline (green and yellow traces respectively in the main panel and in the inset). (iii) The calculated net magnetic field (black) resulting from the superposition of the components shown in (ii) is compared with the data (gray) obtained from one muscle (mean ± SE; n = 450 trials). The best agreement between theory and experiments was obtained for *σ*_*i*_ = 1.5 *S/m, σ*_*p*_ = 0.01 *S/m, σ*_*z*_ = 4.5 *S/m* and *σ*_*s*_ = 3 *S/m*.

The mouse skeletal soleus muscle was modeled as a bundle of N = 887 cylindrical fibers of 40 μm diameter and 1 cm length, according to morphometric studies^18^,^19^. The interstitial space was set to 10 μm^20^,^21^. Each muscle fiber was endowed with passive and active conductances characterized with voltage-clamp recordings on *Xenopus* culture myocytes, and then adjusted in order to reproduce the AP shape recorded in mouse (see Supplementary Fig. 1a and Supplementary information). The central compartment of each cell was provided with a mono-exponential excitatory synapse. Simulations reproduced the AP shape and its symmetric propagation from the center toward the two ends of the cable (Fig. 4a(i)). The axial resistance was set to 80 Ωcm in order to reproduce the temporal pattern of the recorded magnetic signal (peak-to-peak time lapse of ~2 ms). This value of the axial resistance is in the range of estimated cytoplasmatic resistance^22^ and reproduce the experimentally observed AP velocity (Fig. 4a(i)). Figure 4a illustrates the membrane potential over the length of the fiber at different times (i), and the corresponding axial currents flowing along the opposite internal gradients on the two sides of each AP (ii).

Even if transmembrane currents do not contribute to the generation of a detectable MF (see section Magnetic Recordings), they give rise to the currents flowing in the extracellular medium^23^, which partially screen the MF due to intracellular current. In the muscle, where the fibers are closely packed and the extracellular currents are confined to flow along the fiber in an interstitial space of a few micrometers, this screening effect can be strong. In order to calculate the MF generated by the muscle, we used the analytical approach developed by Roth & Wikswo^24^ and we generalized it to the case of a muscle composed of many fibers (see Supplementary information for details). This method presents the advantage of using the Ampere’s law which permits to disentangle the contributions to the MF due to the currents present in each region of the system: the fibers, the bundle, the sheath of connective tissue and the bath (Fig. 4b(i)). The parallel organization of the muscle is mimicked in the model with different conductivities of the extracellular space along the radial (ρ) and the axial directions (z). Membranes limit the diffusion along the radial direction while interstitial space favors diffusion along the axial direction. The other regions have isotropic conductivities. Since a majority of the parameters of the system in Fig. 4b(i) is fixed by the specific experimental condition (see Supplementary information for details), the number of free parameters is reduced to the conductivities of the bundle, *σ*_*ρ*_ and *σ*_*z*_, and the conductivity of the sheath, *σ*_*s*_. Increasing *σ*_*z*_ and/or decreasing *σ*_*p*_ in a range of biologically plausible values constrains the extracellular currents to flow more radially and less axially, hence reducing the punctual net MF at 30 μm from the muscle surface (Supplementary Fig. 2a). Changing the sheaths conductivity had almost no effect (Supplementary Fig. 2b).

As mentioned in section ‘GMR-based sensors’, the local MF source is spatially averaged over the length of the probe. When averaged over the probe’s length, the MF was reduced of about one third and slightly broader in time (Supplementary Fig. 2c). Taking into account this effect, the calculations showed that the currents flowing in the bundle produced a MF (blue trace in Fig. 4b(ii)) that screens about 75% of the MF produced by the currents flowing in the fibers (Fig. 4b(ii) red trace), while the screening due to the currents in the sheath and saline (Fig. 4b(ii) green and yellow traces) were two/three orders of magnitude smaller. The screening is the strongest for the central fibers and weaker for the more superficial (Supplementary Fig. 2d). The calculated net MF is shown in Fig. 4b(iii) (black) and compared to the recorded signal (gray): the agreement between the theory and experiments is excellent both in amplitude and temporal pattern, for a set of biologically plausible values.

## Discussion

In this work we demonstrated that GMR-based technology can be used to design specific magnetic sensors for biological recordings. In order to validate this technology, we proceeded to the detection of the MFs associated with the population AP activity in an *ex-vivo* mouse skeletal muscle. The simplicity of the muscle organization and the high synchronicity of its electrical activity generate a simple, predictable, and large MF. The agreement between experiments and theory thus represents a solid validation of the GMR technology, all the more robust as the experimental system is simple. The bi-phasic pattern of the magnetic signal matched the temporal evolution expected from simple considerations based on AP shape and propagation speed obtained by the electrophysiological recordings. The peak-to-peak amplitude was of the order of a few nT and matched the theoretical prediction of the model based on the electrophysiological characterization of the muscle cell performed here. Our study validates the suitability of GMR-based sensors for biological applications and confirms that the non-isopotential nature of the cable structure generates the intra- and extracellular currents responsible for the net MF, and that the transmembrane currents contribution is weak. Our model suggests that the local net magnetic field is a superposition of contributions coming from both intra- and extra-cellular currents, the latter screening more than half of the intracellular primary sources. The experimental validation of these principles, applicable to cable-like structures such as neurons in the brain, is the first unavoidable step to validate the use of GMR-based sensors in the identification of the cellular generators of the macroscopic MEG signal.

Some attempts have been done by Amaral *et al*.^25^ to use the GMR technology for biomagnetism detection, without being able to achieve a purely magnetic recording, as acknowledged by the authors. Indeed, variations of the extracellular potential are of several orders of magnitude higher than the variation of tension in the sensor induced by a MF of the order of nT. Hence, magnetic recordings can be contaminated by an electrical component when the insulation is not sufficient. By measuring the sensor potential in absence of feeding current (Fig. 3c), we showed here that our electrical insulation was sufficient to obtain a pure magnetic recording. The ~3 nT signal obtained here for the muscle magnetic AP is three orders of magnitude lower than Amaral *et al*. but comparable to that measured in worm with NV centers in diamond^5^. A 3 nT amplitude is just above the upper bound of the predictions^26^,^27^ and the estimates^28^,^29^ for the central nervous system, *i.e.* 0.1 to 1 nT. The large internal potential gradient offered by the AP, the strong synchronicity among muscle fibers, their large diameter and their simple parallel organization, all together can explain that the local magnetic fields we found in the skeletal muscle were slightly larger than the estimates done in the central nervous system.

To date, NV quantum defects and GMR are the two physical effects used for local magnetic recordings on biological preparations. These approaches present comparable signal-to-noise ratio but differs in their perspectives of use. While the technique based on the NV quantum defects is an imaging technique which requires a specific microscopy setup^5^, the GMR-based sensor is a self-contained tool as simple to use as an electrode. As electrodes, GMR sensors are highly malleable in shape. This permits, as shown here, to build multi-sensors probes for simultaneous local recordings in different sub-regions of the biological tissue. More sensitive spin electronics devices, in particular based on magnetic tunnel junctions^30^,^31^, are under development and will allow to drastically reduce the averaging necessary to observe weak biological MFs. No specific shielding was needed for the large signals recorded on the skeletal muscle, but shielding is envisaged for lower magnitudes detection, to avoid ambient noise and to reach the intrinsic limits of the probe. Furthermore, since this technology uses silicon substrate, it is naturally compatible with Complementary Metal Oxide Semiconductor (CMOS) and could integrate the readout electronics^32^. Finally, electrodes and magnetic sensors can be mounted on the same support to allow simultaneous electrical and magnetic detection. We are currently aiming at a miniaturization to the μm scale and mounting on sharp support to develop probes able to penetrate the biological tissue. Intended for *in vitro* and *in vivo* experiments, these sharp probes will allow to perform multiple local recordings in deep structures of the brain, with sensitivity orientations adapted to the specific organizations of the structures of interest. Local recordings will provide the opportunity to establish the relationship between the local sources and the macroscopic MEG signal, whose interpretation is based on hypothesis that have never been the subject of experimental validation.

## Methods

### Giant Magneto-Resistance effect

In ferromagnetic materials, conduction electrons that travel through the lattice experience spin-dependent scattering. According to the spin direction, the density of states at the Fermi level is different, which results in stronger scattering and thus shorter mean-free path for minority-spin electrons. Therefore the conductivity will be higher for electrons exhibiting spins parallel to the magnetization of the material and it will be lower for electrons with spins that are antiparallel^33^.

As the spin diffusion length is much more important than the mean-free path, which is itself longer than the thickness of the layers^34^, the GMR effect can be observed in both CIP (current in plane, Supplementary Fig. 1b) and CPP (current perpendicular to plane, Supplementary Fig. 1a) configurations. All sensors used for the experiments were contacted in CIP configuration as it implies a much more straightforward fabrication process.

### Probe fabrication process

The GMR stack is deposited by sputtering on a 700 μm-thick silicon substrate insulated by a SiO2 layer of 1 μm. The GMR stack has the following composition starting from the top (thicknesses indicated in nm):

Ta(3)/PtMn(18)/CoFe(2)/Ru(0.85)/CoFe(2.1)/Cu(2.3)/CoFe(1.5)/NiFe(3.5)/Ta(3)/SiO2/Si.

The pinned layer is composed of an antiferromagnet (PtMn) coupled to a synthetic antiferromagnet (CoFe/Ru/CoFe)^35^, while the soft layer is made of a bilayer of CoFe (high spin polarization) coupled to NiFe (very low coercivity). The GMR stack is patterned by laser lithography. The GMR segments are etched by ion milling under Argon gas at 10^-4^ mbar. Contacts consist of a bilayer of Ti (15 nm)/Au (150 nm) deposited by electron beam evaporation at 10^-8^ mbar in a lift off process. Each of the three segments is contacted at its ends (Fig. 2b). The current is injected along the segment length in the plane of the stack. The whole structure, except for the contact pads opposite to the segments is insulated by a 300 nm Al_2_O_3_/Si_3_N_4_ bilayer deposited by sputtering and topped by a 15 μm layer of polymer resin to ensure insulation of the sensors submerged in the physiological saline solution. The polymer resin layer was necessary to ensure a proper insulation. Indeed, in absence of polymer resin, an electrical contamination corresponding to a voltage variation in the GMR much more important than the variations induced by the biological signal was present in absence of feeding current. Finally, laser cutting was used to define the square shape of the overall probe. The probe was glued on a printed circuit board (PCB) and contacted to the copper lines by wire-bonding. Wire-bonds (30 μm thick) were protected by encapsulation in araldite glue. We made a hollow Teflon chamber in which a 5 mm thick layer of silicone was poured to handle easily the needles used to fasten the soleus by its tendons. A biasing ferrite magnet was taped under the chamber to keep the magnetization of the free layer orthogonal to the pinned layer direction in absence of an applied field. This allows linearization of the sensor response around zero field^36^.

### Transport characterization

The three GMR segments were characterized by magneto-transport and noise spectral density measurements at room temperature. A DC current of 1 mA was applied to the each segment. The output voltage was amplified and low-pass filtered at 30 Hz (Stanford Research SR560). Helmholtz coils were used to provide a uniform magnetic field that covers the range of sensitivity of the three spin valves along the sensing direction. The sensor performances are estimated through their sensitivity and their noise. The magnetoresistance ratio MR is defined by: MR = 2(R_AP_ − R_P_)/(R_AP_ + R_P_), where R_AP_ and R_P_ are the resistances of the spin valve for the anti-parallel and parallel states of magnetization respectively. The sensitivity of the probe corresponds to the slope of the R(H) relationship. It is expressed in V/V/T. Supplementary Figure 2 shows the output voltage variation as function of an applied in-plane field (along the pinned layer direction) for the three segments. The MR ratio is 6.4% for segment 1, 6.5% for segment 2 and 6.6% for segment 3. The sensitivity around zero field of the different probes used for this study is typically varying between 37 V/V/T and 75 V/V/T according to the segment and its magnetic history.

The experiments have been performed with a 20 mA feeding current, corresponding to 1.6 V on the segment. In order to ensure proper calibration of sensitivity the output signal is measured prior to the recordings with a calibrated signal generated by an air coil. As an example, the probe used in Fig. 3b exhibited a sensitivity of 85 V/T in segment 1, of 60 V/T in segment 2 and 120 V/T in segment 3.

### Noise measurements

GMR devices are subject to fluctuations, among which the main components are the thermal noise and the 1/f noise^37^. Noise spectral density measurements were performed in a magnetically shielded room. Voltage supply was provided by two 9 V batteries, connected to a Wheatstone bridge that includes the GMR sensor and an adjustable resistance. Both outputs were sent in a differential mode to a low noise amplifier (INA 103). The amplifier output was amplified and filtered (Stanford Research 560 fed on battery) (Supplementary Fig. 3). The total gain of the acquisition line was typically between 10.000 and 100.000 for a bandwidth of [0.1 Hz; 10 kHz]. An external magnetic field, in the μT range at 30 Hz, generated by an air coil, was used for calibration purposes. The Power Spectral Density (PSD) of the noise floor due to thermal fluctuations is given by: 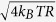 (in units of V/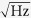), where k_B_ is the Boltzmann constant, T the temperature and R the resistance. It leads to 1.15 nV/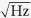 at room temperature for a GMR segment of 80 Ohms. The off-line 1.2 kHz low-pass filtering leads to an integrated noise of 3.5 nT rms (root mean square) for an approximated field noise of 100 pT/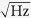 in this working bandwidth. As the magnetic signature had an average peak-to-peak amplitude of 2.8 nT, averaging the signal over 500 events allows reaching a maximum SNR of 16.

### Electrophysiology

Animal care followed the European Union regulations (O.J. of E.C. L358/1 18 December 1986), and the European directive 2010/63/UE. 3- to 5-month-old Swiss mice were anesthetized with isoflurane, and cervical dislocated. Dissections were performed within 15 min in an oxygenated Ringer solution of the following composition (in mM): 145 NaCl, 3 KCl, 2 CaCl2, 1 MgCl2, 10 HEPES (pH 7.4) and 11 glucose. Intra- and extra-cellular recordings were performed at room temperature in the oxygenated Ringer solution defined above. Sharp pipettes were made from borosilicate glass (Clark Electromedical Instruments), pulled on a P-1000 puller (Sutter Instrument Company), and had a resistance of 40–60 M when filled with a KCl 3 M solution. The filled pipette tip was cut at the limit of the pulled zone, and used as floating electrode^9^. The chlorided end of a 10 cm long, 50 μm diameter, silver wire was introduced inside this electrode, and plugged by its opposite end to the head-stage of a SEC 0.5X amplifier (NPI electronic GmbH, Tamm, Germany), where it hung loosely above the muscle. A drop of mineral oil was added at the back of the intra-pipette medium to avoid evaporation. The pipette pendulum was vertically manipulated to enter the muscle cells, and its flexibility allowed stable membrane potential recordings in contracting muscles. Nerve APs were induced with 6 V, 30 μs voltage steps in a suction pipette.

#### Data acquisition

Magnetic recordings were done in the same conditions than electrophysiological recordings. Electrical and magnetic signals were digitized by a 16 bit A/D converter (Digidata 1322A, Axon Instruments, Union City, CA, USA) and acquired with the PClamp9 software (ibid). Magnetic recordings were off-line filtered with a 80–1200 Hz bandwidth.

## Additional Information

**How to cite this article**: Barbieri, F. *et al*. Local recording of biological magnetic fields using Giant Magneto Resistance-based micro-probes. *Sci. Rep.* **6**, 39330; doi: 10.1038/srep39330 (2016).

**Publisher's note:** Springer Nature remains neutral with regard to jurisdictional claims in published maps and institutional affiliations.

## Supplementary Material

Supplementary Information
