# Supplementary material for: Local recording of biological magnetic fields using Giant Magneto Resistance-based micro-probes
Source: Sci Rep. 2016 Dec 19;6:39330. doi: 10.1038/srep39330 (PMC5171880; doi:10.1038/srep39330)
Supplement: Supplementary Information [file srep39330-s1.pdf]

Local recording of biological magnetic fields using Giant Magneto Resistance-based micro-probes.

Francesca Barbieri, Vincent Trauchessec, Laure Caruso, Josué Trejo-Rosillo, Bartosz Telenczuk, Elodie Paul, Thierry Bal, Alain Destexhe, Claude Fermon, Myriam Pannetier-Lecoeur, Gilles Ouanounou

Supplementary Information

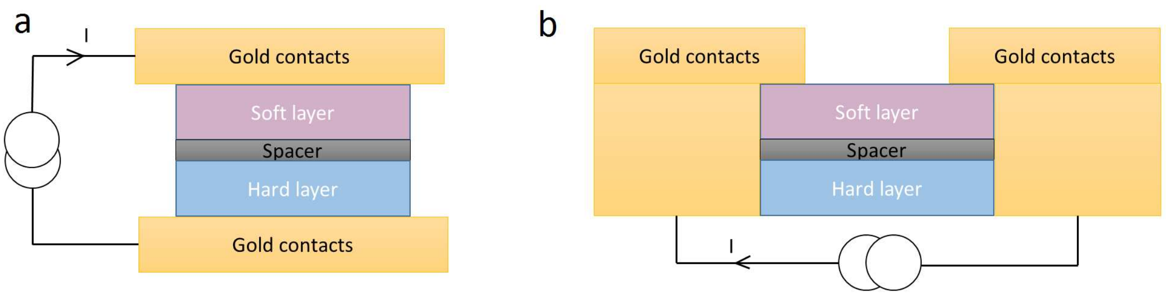

Supplementary Figure 1: **Configuration of GMR connexions**. **a** Current perpendicular to the plane (CPP) configuration. **b** Current in plane (CIP) configuration used during the probe fabrication process.

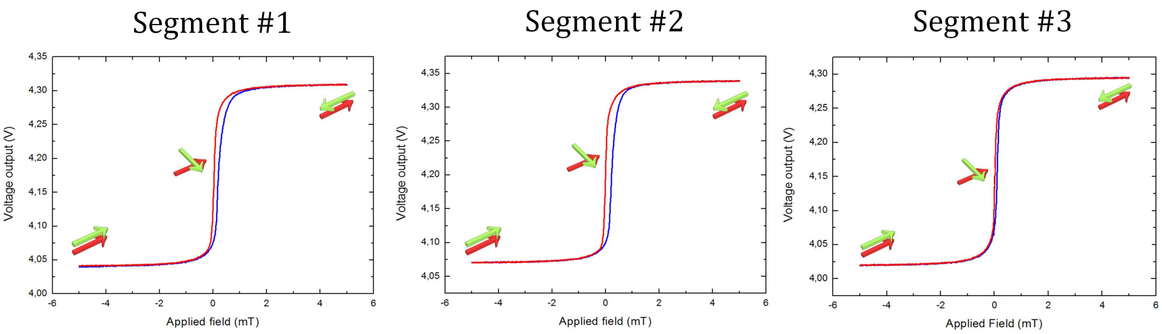

Supplementary Figure 2: Voltage variation of each of the three segments as a function of an in-plane field applied along the pinned layer magnetization.

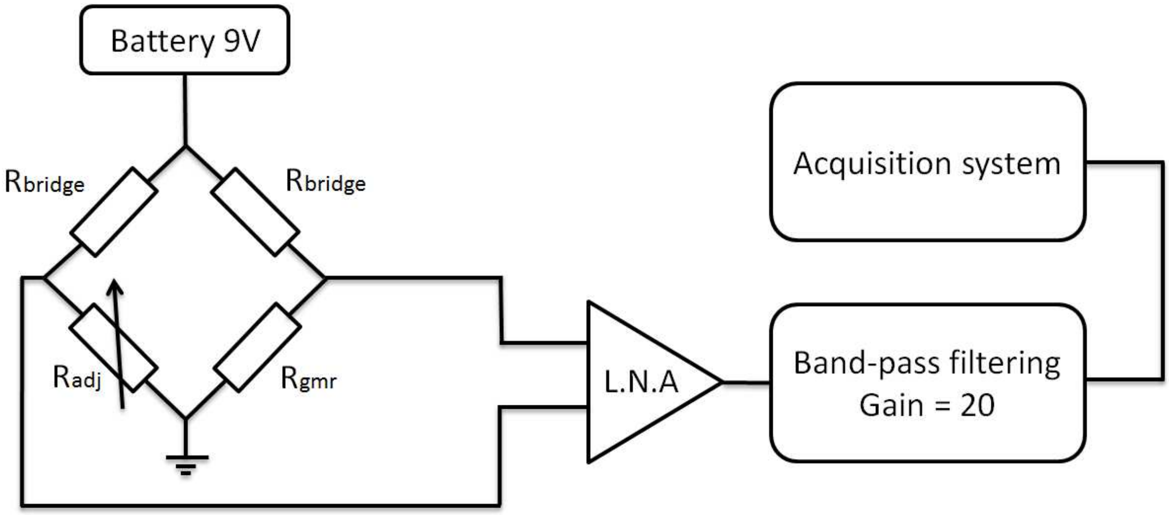

Supplementary Figure 3: Setup used for DC measurements, with $R_{bridge} = 500\Omega$. The value of R_{adj} is set to the same value as R_{gmr} so that the differential output of the Wheatstone bridge is close to 0 V, in order to only amplify the variations of R_{gmr}.

Model

Single fiber model. The muscle cell was modeled as a cylindrical cable composed of 1000 compartments of $10\mu m$ length and $40\mu m$ diameter. Because of poor space-clamp, voltage-clamp recordings in soleus skeletal muscle were difficult. For this reason, we performed the voltage-clamp characterization of muscle cell currents in cultured xenopus myocytes and of synaptic currents in the flexor-digitorum-brevis (FDB) of the mouse. We then ajusted the parameters that characterize the different currents in order to reproduce the AP shape recorded on the soleus skeletal muscle cells under floating electrode recording conditions. All simulations were performed with NEURON (Hines and Carnevale 1997).

Procedure to characterize soleus skeletal muscle currents. We combined the results obtained from the myocyte voltage-clamp recordings with recordings of synaptic conductance and EPSP in the flexor-digitorum-brevis (FDB) of the mouse, in order to reproduce the AP recorded in the soleus skeletal muscle. We proceeded as follows:
Synaptic current. The synaptic current was modeled as a current of the form: $I_{syn} = g_{syn}(t)(V - E_{syn})$, with reverse potential, $E_{syn} = 0$, and synaptic conductance of the form, $g_{syn}(t) = g_{syn,max} \exp(-t/\tau_{syn})$. The synaptic decay time constant was fitted from recordings of synaptic conductance of the FDB under voltage-clamp and set to $\tau_{syn} = 0.58ms$. Since the maximal conductance, $g_{(syn,max)}$, recorded in the FDB was not sufficient to elicit an AP in our model fiber, we choose to set the maximal synaptic conductance at the minimal value necessary to elicit an AP, which was $g_{(syn,max)} = 10\mu S$.
Kir and leak currents. The characteristics of the Kir current were obtained from the myocyte voltage-clamp study. Myocyte Kir current was expressed by the form:$I_{Kir} = g_{max} m(V - E_K)$, where the maximal conductance g_{max} is set to $600\mu S/cm^2$, and the variable m evolves in time according to:

$$m(t) = m_\infty - (m_\infty - m_0)e^{t/\tau_m} \tag{1}$$

where m_∞ and τ_m where determined through fitting of the isolated Kir current, as:

$$m_\infty = \frac{1}{1 + \exp(-0.074(-91.6 - V))} \tag{2}$$

and $\tau_m = 0.2ms$ The leak current was then adjusted in order to reproduce the decay dynamics of the EPSP recorded in FDB voltage-clamp recordings (Fig. 4b). The leak current was modeled as: $I_{leak} = g_{leak}(V - E_{leak})$, where $g_{leak} = 200\mu S/cm^2$ and $E_{leak} = -90mV$.
Sodium and potassium currents. After determining the synaptic, Kir and leak currents parameters, we adjusted the parameters of sodium and potassium currents in order to fit the average AP recorded during floating electrode recordings on the soleus skeletal muscle (Fig. 4a). Since the average baseline membrane potential was slightly depolarized due to pipette leak, we added in the compartment corresponding to the recording site, a pipette

leak current of conductance $g_{pipette} = 43\mu S/cm^2$ and reversal potential $E_{pipette} = 0$.After adding the pipette leak, we adjusted the sodium and potassium currents that were modeled as follows:

$$I_{Na} = g_{(Na,max)} m^3 h (V - E_{Na}) \tag{3}$$

with $g_{Na,max} = 0.028 S/cm^2$ and $E_{Na} = 50mV$ and:

$$m_\infty = \frac{1}{1 + \exp\left(0.43(-37.7 - V)\right)} \tag{4}$$

$$\tau_m = 0.12 \exp\left(-0.01354(V + 55)\right) \tag{5}$$

$$h_\infty = \frac{1}{1 + \exp\left(-0.8(-50 - V)\right)} \tag{6}$$

$$\tau_h = 0.48 \exp\left(-0.01252 * (V + 22)\right) \tag{7}$$

$$I_{(K,TEA)} = g_{(K,TEA,max)} n^4 (V - E_K)$$

with $g_{(K,TEA,max)} = 0.02 S/cm^2$ and $E_K = -90mV$ and:

$$n_{(\infty,TEA)} = \frac{1}{1 + \exp\left(0.06(-30 - V)\right)} \tag{8}$$

$$\tau_{(n,TEA)} = 1.6 \exp\left(-0.005(V + 20)\right) \tag{9}$$

$$I_{(K,4AP)} = g_{(K,4AP,max)} n^4 (V - E_K)$$

with $g_{(K,4AP,max)} = 0.2 S/cm^2$ and $E_K = -90mV$ and

$$n_{(\infty,4AP)} = \frac{1}{1 + \exp\left(0.08(-36 - V)\right)} \tag{10}$$

$$\tau_{(n,4AP)} = 1.6 \exp\left(-0.0193(V - 79)\right) \tag{11}$$

Axial intracellular currents. In section 'Magnetic Recordings', we showed that trans-membrane currents do not generate a detectable MF. This result was not unexpected both because transmembrane currents generates opposite contributions to the MF, thanks to the quasi-homogeneous distribution of channels on the membrane surface and because the MF produced by trans-membrane currents is expected to be negligible compared to that of axial currents (Woosley et al. 1985; Hamalainen et al. 1993). On the contrary, currents

flowing along the muscle axis were found to be the primary generators of the recorded MF.

The axial intacellular currents in each compartment i, were calculated as

$$I_i = \frac{(V_{i+1} - V_i)}{R_i} \tag{12}$$

where V_i and R_i are respectively the membrane potential and the axial resistance of compartment i. R_i can be expressed in term of specific resistivity ρ as:

$$R_i = \frac{\rho\, l}{A} \tag{13}$$

where l and A are length and cross section of the compartment.

Even if transmembrane currents do not contribute to the generation of a detectable MF, they are at the origin of the currents flowing in the extracellular medium (Einevoll et al. 2013). As shown in the scheme of Figure 1a, if a local source and sink of current (in this example a depolarizing sodium current and a leak current) are present at two different sites along the cable, local charge variations in the extracellular medium create a potential gradient with opposite polarity with respect to the intracellular space. Hence, extra- and intracellular gradients generates currents in opposite directions. Because of the small extracellular resistivity, extracellular currents are generally smaller than intracellular currents and dispersed, at least in the cortex, in a larger volume. On the contrary, inside of the muscle, the fibers are closely packed and the extracellular currents are confined to flow along the fiber in an interstitial space of a few micrometers. Since the axial resistance is inversely proportional to the cross section (Eq. 13), small cross section means high axial resistance, which in turn translates to larger potential gradients. As shown in section 'Electrophysiological Recordings', when APs were triggered in the muscle, a potential with a peak-to-peak amplitude of around 6 mV was recorded in the extracellular medium. Extracellular currents in the muscle are then likely to contribute considerably to the generation of the MF.

Calculation of the magnetic field generated by the soleus skeletal muscle. The magnetic field produced by an AP travelling in a single muscle cell was calculated using the approach developed by (Roth and Wikswo 1985) for an axon in a nerve bundle, and we generalized it to the case of a muscle composed by several fibers. For the sake of clarity, we summarize here the main lines of the method and we describe how the calculations were generalized for the case of the entire muscle.

The geometry of the muscle is depicted in figure 4c. A cylindrical fiber of diameter, $a = 40\mu m$, is located at a distance, t, from the center of the bundle. The bundle has a diameter, $b = 170\mu m$, which was the resulting diameter of $N = 887$, 40 μm diameter fibers separated by a 10 μm interstitial space and it is surrounded by a sheath of thickness, $\delta = 10\mu m$. Two coordinates systems are necessary to describe the bundle: the primed system which is centered at the fiber, and an unprimed system centered at the bundle. The two systems are related by simple relationships (Roth and Wikswo 1985).

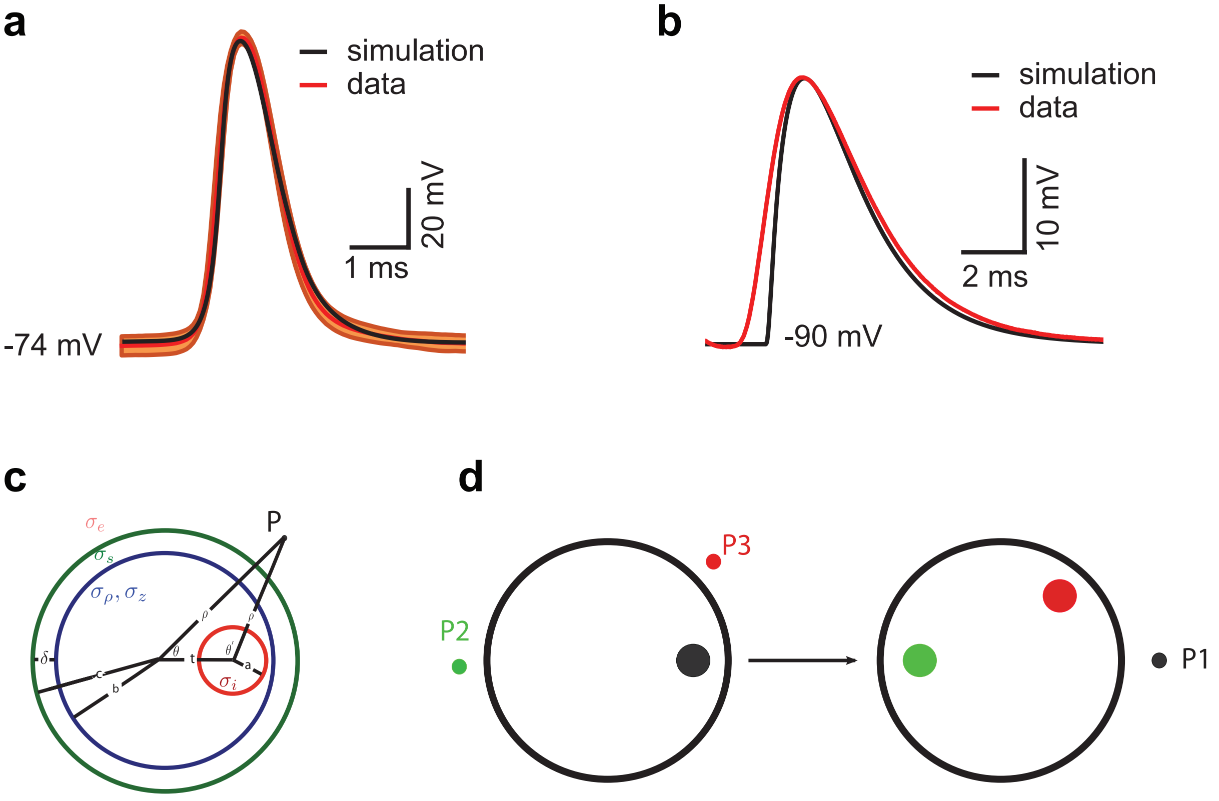

Supplementary Figure 4: **a** Comparison between the recorded (red, mean ± SD, n=6) and the simulated (black) action potential. **b** Comparison between the EPSP produced by synaptic stimulation of the FDB cholinergic synapse (red) and the EPSP in the fiber model due to one synaptic input (black). **c** Scheme of the muscle model. **d** Scheme to explain the approximation used in the determination of the MF of the entire muscle (see text).

The interior of the fiber and the physiological saline had homogeneous, isotropic conductivities σ_i and σ_e. In order to mimick both the presence of the others fibers, that limit the diffusion of the currents along the radial direction, and the interstitial space, that, instead, favours the diffusion of the currents along the axial direction in the near surrounding of the fiber surface, the bundle itself was modeled as an anisotropic medium, with different conductivities along the radial and axial directions, σ_ρ and σ_z. The bundle itself was then surrounded by a thin sheath of connective tissue with conductivity σ_s.
The calculation of the MF passes first through the calculation of the potential in each of the regions of the system. This can be done by solving Laplace's equation with suitable boundary conditions. Since we know from the simulation of the AP dynamics, the value of the transmembrane potential along the fiber at every point in time, the first boundary condition consists in assuming that the potential at $\rho^{'} = a$ is equal to the membrane potential, $\phi_m(z)$, at each point z along the fiber. Furthermore, the potential has to be continuous at the boundaries, $\rho = b$ and $\rho = c$, and the normal component of the current density has to be continuous across all three interfaces. The potential in the bundle, which is anisotropic, also solves the Laplace's equation provided the following coordinate transformation: $\rho^* = \sqrt{(\sigma_z/\sigma_\rho)}\rho$.
The potential in the four regions can be written in the Fourier space as expansions in the eigenfunctions of the Laplace's equation in cylindrical coordinates, i.e. in terms of modified Bessel functions and Fourier sum of trigonometric functions,

$$\phi_i(\rho^{'}, \theta^{'}, k) = A_0(k) I_0(|k|\rho^{'}) + 2\sum_{m=1}^{\infty} A_m(k) I_m(|k|\rho^{'}) cos(m\theta^{'}) \tag{14}$$

$$\begin{aligned}\phi_b(\rho^{'*}, \theta^{'}, k) = {} & B_0(k) I_0(|k|\rho^{'*}) + C_0(k) K_0(|k|\rho^{'*}) \\ & + 2\sum_{n=1}^{\infty} (B_n(k) I_n(|k|\rho^{'*}) + C_n(k) K_n(|k|\rho^{'*})) cos(n\theta^{'})\end{aligned} \tag{15}$$

$$\begin{aligned}\phi_s(\rho, \theta, k) = {} & D_0(k) I_0(|k|\rho) + E_0(k) K_0(|k|\rho) \\ & + 2\sum_{m=1}^{\infty} (D_m(k) I_m(|k|\rho) + E_m(k) K_m(|k|\rho)) cos(m\theta)\end{aligned} \tag{16}$$

$$\phi_e(\rho, \theta, k) = F_0(k) K_0(|k|\rho) + 2\sum_{m=1}^{\infty} F_m(k) K_m(|k|\rho) cos(m\theta) \tag{17}$$

where k denotes the spatial frequency and $A_m(k)$, $B_m(k)$, $C_m(k)$, $D_m(k)$, $E_m(k)$ and $F_m(k)$ are unknown coefficients that have to be determinated from the boundary conditions. As mentioned above, there are six boundary conditions: one, at $\rho^{'} = a$, imposes that

the Fourier transform of the membrane potential is equal to the difference between the intracellular and bundle potential, $\phi_m(k) = \phi_i(a, \theta', k) - \phi_b(a, \theta', k)$. The others five conditions are given imposing the continuity of the potential at $\rho = b$ and $\rho = c$ and of the normal component of the current density across all three surfaces. The full expressions of these boundary conditions are detailed in (Roth and Wikswo 1985) and were solved using standard Python routines (available in NumPy package) for a system of linear scalar equations. We used $m = 6$ terms to approximate the infinite series in equations 14-17.

Once the expressions of the potential are obtained, the current density can be calculated by differentiating equations 14-17 and through the equality $J = -\sigma \nabla \phi$. From the current density (Roth and Wikswo 1985), one calculates the azimuthal component of the MF averaged over all angle θ using the Ampere's law. The integration over θ eliminates all terms in the expression of the current density as a Fourier sum except the $m = 0$ term, hence greatly simplifying the calculations. The integration performed over each region of the system gives the expression of the magnetic field in the Fourier space, B, as follows:

$$B(\rho, k) = B_i(\rho, k) + B_b(\rho, k) + B_s(\rho, k) + B_e(\rho, k) \tag{18}$$

where

$$B_i(\rho, k) = i\frac{\mu_0 \sigma_i k a}{\rho |k|} A_0(k) I_1(|k|a). \tag{19}$$

$$B_b(\rho, k) = B_{b1}(\rho, k) + B_{b2}(\rho, k) + B_{b3}(\rho, k) \tag{20}$$

where

$$\begin{aligned} B_{b1}(\rho, k) = i\frac{\mu_0 \sigma_z k}{\rho|k|}\sqrt{\frac{\sigma_\rho}{\sigma_z}}\Bigg[\Bigg[B_0(k) I_0\Bigg(|k|\sqrt{\frac{\sigma_z}{\sigma_\rho}}t\Bigg) + 2\sum_{n=1}^{\infty} B_n(k) I_n\Bigg(|k|\sqrt{\frac{\sigma_z}{\sigma_\rho}}t\Bigg)\Bigg] \\ + \Bigg[C_0(k) K_0\Bigg(|k|\sqrt{\frac{\sigma_z}{\sigma_\rho}}t\Bigg) + 2\sum_{n=1}^{\infty} C_n(k) K_n\Bigg(|k|\sqrt{\frac{\sigma_z}{\sigma_\rho}}t\Bigg)\Bigg]\Bigg] t I_1\Bigg(|k|\sqrt{\frac{\sigma_z}{\sigma_\rho}}t\Bigg) \end{aligned} \tag{21}$$

$$\begin{aligned} B_{b2}(\rho, k) = i\frac{\mu_0 \sigma_z k}{\rho|k|}\sqrt{\frac{\sigma_\rho}{\sigma_z}}\Bigg[\Bigg[B_0(k) I_0\Bigg(|k|\sqrt{\frac{\sigma_z}{\sigma_\rho}}t\Bigg) + 2\sum_{n=1}^{\infty} B_n(k) I_n\Bigg(|k|\sqrt{\frac{\sigma_z}{\sigma_\rho}}t\Bigg)\Bigg] \\ \times \Bigg[b I_1\Bigg(|k|\sqrt{\frac{\sigma_z}{\sigma_\rho}}b\Bigg) - t I_1\Bigg(|k|\sqrt{\frac{\sigma_z}{\sigma_\rho}}t\Bigg)\Bigg] \\ + \Bigg[C_0 I_0\Bigg(|k|\sqrt{\frac{\sigma_z}{\sigma_\rho}}t\Bigg) + 2\sum_{n=1}^{\infty} C_n(k) I_n\Bigg(|k|\sqrt{\frac{\sigma_z}{\sigma_\rho}}t\Bigg)\Bigg] \\ \times \Bigg[t K_1\Bigg(|k|\sqrt{\frac{\sigma_z}{\sigma_\rho}}t\Bigg) - b K_1\Bigg(|k|\sqrt{\frac{\sigma_z}{\sigma_\rho}}b\Bigg)\Bigg]\Bigg] \end{aligned} \tag{22}$$

$$B_{b3}(\rho,k) = -i\frac{\mu_0\sigma_z k}{\rho|k|}\sqrt{\frac{\sigma_\rho}{\sigma_z}}\Bigg[B_0(k)aI_1\Bigg(|k|\sqrt{\frac{\sigma_z}{\sigma_\rho}}a\Bigg) + C_0(k)\Bigg[\frac{1}{|k|}\sqrt{\frac{\sigma_\rho}{\sigma_z}} - aK_1\Bigg(|k|\sqrt{\frac{\sigma_z}{\sigma_\rho}}a\Bigg)\Bigg]\Bigg] \tag{23}$$

$$B_s(\rho,k) = i\frac{\mu_0\sigma_s k}{\rho|k|}(D_0(k)(cI_1(|k|c) - bI_1(|k|b)) + E_0(k)(bK_1(|k|b) - cK_1(|k|c))). \tag{24}$$

$$B_e(\rho,k) = i\frac{\mu_0\sigma_e k}{\rho|k|}F_0(k)(cK_1(|k|c) - \rho K_1(|k|\rho)). \tag{25}$$

The integration over all angles θ was justified in the work of (Roth and Wikswo 1985), because, in their case, the MF produced by an axon was considered to be measured by a toroid encircling the nerve bundle. Here, we want to calculate the azimuthal component of the field in a point at distance ρ and angle $\theta = 0$ from the center of the bundle, hence, in principle, the averaging over θ should not be appropriate. However, if we consider a set of N_t fibers equally distributed on a circle of radius t inside the bundle, we can approximate the MF generated by the N_t fibers in a point P1 as $N_t\langle B_t\rangle$, where $\langle B_t\rangle$ is the θ-averaged MF of a single fiber at distance t from the center (i.g. the black fiber in figure 4d (left). This is because, averaging the MF of a single fiber across θ means summing the contributions of the MF at every point on a given circle around the muscle, as, for example, points P2 and P3 of figure 4d (left). However, the MF generated by the black fiber in P2 and P3 is equivalent to the MF generated in P1 by respectively the green and red fibers depicted in figure 4d (right). This fact being true for all fibers N_t, permits to approximate the MF of the entire muscle, composed by N fibers, as $\sum_{\{t\}} N_t\langle B_t\rangle$. This approximation is as more precise as t is close to the bundle radius, because the most superficial layers have an higher N_t. However, because of the dependency of the screening on the position of the fiber (see below and Fig. 5d), 90% of the signal is due to the five more superficial layers which have large values of N_t. Hence, we can consider $\sum_{\{t\}} N_t\langle B_t\rangle$ as a resonable first approximation of the entire MF.

The method of (Roth and Wikswo 1985) presents the advantage of using the Ampere's law which permits to calculate separately the contributions to the MF due to the currents present in each of the regions in Fig. 4b(i). We have seen that the intra- and extracellular currents flow in opposite directions. Since currents of opposite direction produce MF of opposite sign, we can expect that the MF generated by the extracellular currents could screen, at least partially, the MF generated by the intracellular currents. The Ampere's law permits to evaluate the importance of these effects by calculating the contributions

due to the currents flowing inside the fiber, in the bundle, in the sheath and in the external saline.

The number of parameters in the model could be large (values of the conductivities, dimensions, etc). However, most of the parameters in the model were kept fixed at plausible values and we reduced to three the number of free parameters. First, our measurements of MF were performed at an estimated distance of $\sim 30 \ \mu m$ from the surface of the muscle, considering that the muscle was practically in contact with the probe, and that only a passivation layer of $\sim$15 μm separated the sensor from the saline. The value of the saline conductivity was set to typical value for Ringer solution, 1.6 S/m (Koch), while the value of the intracellular conductivity was fixed at 1.25 S/m, in order to reproduce both the AP dynamics (Fig. 4a(i)) and the magnetic signal pattern (Fig. 4b(iii)). Finally, as mentioned above, number and dimensions of fibers, and dimension of the interstitial space were fixed at average values taken from literature. Therefore, the number of free parameters that were varied in the model in order to reproduce the amplitude of the recorded signal, were only the conductivities of the bundle, σ_ρ and σ_z, and the conductivity of the sheath, σ_s.

The MF can be calculated for a single fiber located at different distances, t, from the center of the bundle. This permits to understand the behaviour of the MF due to the different currents of the system as a function of the conductivities. Figure 5d shows the total MF, B_{tot}, perceived in the point P and the relative contributions due to the intracellular current, B_i, the currents flowing in the bundle, B_b, in the sheath, B_s, and in the external saline, B_e. As more as the fiber is far from the internal surface of the bundle (black $\rightarrow$ light gray), as more the currents in the bundle become important and screen the MF generated by the intracellular currents. This screening effect is larger and larger as the ratio σ_z/σ_ρ increases (Fig. 5a). On the contrary, contributions from the currents in the bath and the sheath, are stronger when part of the currents in the bundle can flow outside, i.e when the fiber is closer to the surface. However, contributions from bath and sheath currents are much smaller than that of bundle currents. Hence, extracellular bundle currents can be considered as the primary source of screening.

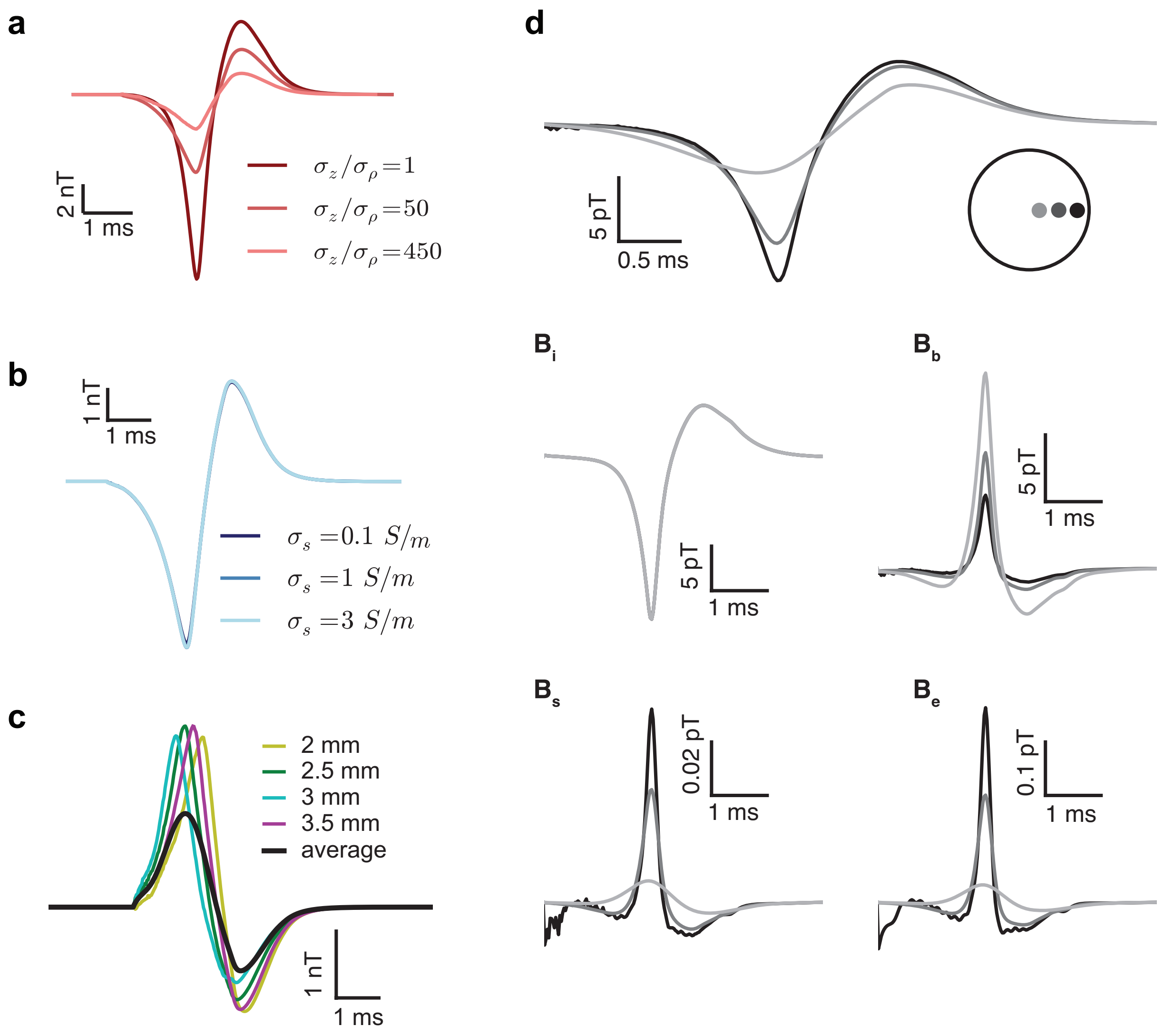

Supplementary Figure 5: **a** Punctual magnetic field generated by the entire muscle at $30\mu m$ from the surface for different values of the ratio σ_z/σ_ρ ($\sigma_s = 3S/m$). **b** Same as in (a), but when varying σ_s ($\sigma_\rho = 0.01S/m$, $\sigma_z = 3S/m$). **c** Effect of the averaging over the probe length: the punctual magnetic field generated at $30\mu m$ from the surface is shown for different position along the muscle (colored traces). The black trace represents the time evolution of the average of the magnetic field over the positions spanning the probes length (1.7 mm). **d** Behavior of the MF of a single fiber depending on its position inside the bundle. Top: net magnetic field. Bottom: The different MF components due to the currents flowing in the fiber (B_i), the bundle (B_b), the sheath (B_s) and the saline (B_e)

References

Einevoll, G. T., C. Kayser, N. K. Logothetis, and S. Panzeri (2013, November). Modelling and analysis of local field potentials for studying the function of cortical circuits. *Nature reviews. Neuroscience 14*(11), 770–85.

Hamalainen, M., R. Hari, R. Ilmoniemi, J. Knuutila, and O. V. Lounasmaa (1993). Magnetoencephalographytheory, instrumentation, and applications to noninvasive studies of the working human brain. *Rev Mod Phys 65*, 413497.

Hines, M. L. and N. T. Carnevale (1997, August). The NEURON simulation environment. *Neural computation 9*(6), 1179–209.

Koch, C. *Biophysics of Computation: Information Processing in Single Neurons.(ed Stryker M.), (New York: Oxford Univ. Press, 1998).*

Roth, B. and J. Wikswo (1985). The electrical potential and the magnetic field of an axon in a nerve bundle. *Mathematical Biosciences 76*(1), 37–57.

Woosley, J., B. Roth, and J. Wikswo (1985). The magnetic field of a single axon: a volume conductor model. *Mathematical Biosciences 76*, 1–36.
